# Genetic Markers and Mutations in Primary Spinal Cord Tumors and Their Impact on Clinical Management

**DOI:** 10.3390/brainsci15101028

**Published:** 2025-09-23

**Authors:** Rouzbeh Motiei-Langroudi

**Affiliations:** Department of Neurosurgery, University of Kentucky, 780 Rose St., MS107A, Lexington, KY 40536, USA; rouzbeh.motiei@uky.edu; Tel.: +1-859-323-5661; Fax: +1-859-323-1330

**Keywords:** spinal cord tumors, intramedullary, ependymoma, astrocytoma, glioblastoma, genetic markers, neuro-oncology

## Abstract

Primary spinal cord tumors are rare neoplasms representing 2–4% of central nervous system tumors. Despite their low incidence, their impact on neurological function is profound. Historically, tumor classification and management have relied primarily on histopathology. However, advances in molecular diagnostics have highlighted the critical role of genetic alterations in tumor behavior, prognosis, and treatment response. This narrative review summarizes current evidence on genetic mutations in primary intramedullary spinal cord tumors, focusing on their prognostic value and implications for clinical management. Emphasis is placed on the integration of genetic features into diagnostic criteria and clinical practice, as distinct molecular profiles define many spinal cord tumor subtypes. Integration of molecular diagnostics into spinal cord tumor management represents a paradigm shift from morphology-based to biology-driven practice. Genetic alterations inform prognosis, refine risk stratification, and increasingly guide therapeutic decision-making, including the use of targeted therapies and adjuvant radiation. Despite progress, challenges remain due to the rarity of these tumors, small sample sizes, and limited access to molecular testing. Ultimately, molecular precision promises to enhance survival and quality of life for patients with these rare but impactful tumors.

## 1. Introduction

Spinal cord tumors represent a diverse group of central nervous system (CNS) neoplasms, classified based on their anatomical location as intradural intramedullary, intradural extramedullary, or extradural [1]. Relatively rare compared to intracranial tumors (2–4% of primary CNS tumors), their impact on neurological function can be profound [2]. While traditional classifications and treatment paradigms have relied heavily on histopathological features, recent advances in molecular diagnostics have revealed that genetic alterations play a crucial role in tumor behavior, prognosis, and therapeutic response [3,4]. The 2021 World Health Organization (WHO) Classification of CNS Tumors introduced a paradigm shift by emphasizing molecular subtypes added to traditional histology, particularly for ependymomas [5].

Molecular diagnostics have transformed the understanding of spinal cord tumors. Techniques such as deoxyribonucleic acid (DNA) methylation profiling, next-generation sequencing (NGS), fluorescence in situ hybridization (FISH), and immunohistochemistry (IHC) have become instrumental in refining diagnoses and guiding treatment. These tools can detect specific gene fusions, mutations, and epigenetic modifications that are not evident through histology alone [6]. This brief review focuses on the primary intramedullary spinal cord tumors with an emphasis on genetic mutation expression and how these influence clinical management. The implications of these markers in radiation and chemotherapy decisions, treatment stratification, and prognostication are also explored.

## 2. Tumor-Specific Genetic Profiles Affecting Behavior and Prognosis

In this section, the major primary spinal cord tumor pathologies are reviewed, focusing on their molecular markers, prognostic significance, and therapeutic implications. A summary is presented in Table 1.

### 2.1. Ependymoma

Ependymomas are among the most prevalent primary spinal tumors and are now classified based on molecular features. The WHO 2021 classification of spinal cord ependymomas includes spinal ependymoma (SP-EPN), spinal ependymoma with *N-Myc* (also known as *MYCN*) amplification (SP-MYCN), myxopapillary ependymoma (MPE), subependymoma (SE), and neurofibromatosis 2 (NF2)-mutated variants (with losses on chromosome 22q). The SP-EPN and the rare SP-MYCN variants are exclusive to the spinal cord, while the others can also happen in supratentorial and posterior fossa compartments [33].

While spine ependymomas (including those with NF2 mutation) are, in general, regarded as good prognosis [34], *MYCN*-amplified spinal ependymomas in particular exhibit aggressive behavior, rapid progression, early metastases, and leptomeningeal dissemination. The recurrence rate is reported to be in the 75–100% range [11]. Therefore, they are associated with poorer prognosis and require multimodal therapy [10]. Both studies on SP-MYCN outcome, however, suffer from a low case volume (cohorts of thirteen and four patients, respectively). On pathology, these possess high-grade features including microvascular proliferation, necrosis, and high mitotic cell count. Diagnosis of pathognomonic high *MYCN* amplification is confirmed by FISH [35,36]. While rare, it emphasizes the importance of DNA methylation profiling and gene fusion detection for classification and guiding treatment options.

MPE is associated with a better outcome and long-term overall survival rates has been reported to exceed 90% [37]. However, approximately 20% are associated with local or distant recurrence. As a result, they were upgraded to WHO grade 2 in the 2021 classification. While not bearing immediate prognostic data, the homeobox gene *HOXB13* (which is often seen in prostate cancer) has emerged as a useful immunohistochemical marker for distinguishing MPE from other spinal ependymomas, as shown in a study including 143 spinal neoplasms (54 MPE, 46 spinal ependymomas, and various other tumor types) [13].

Another study in 37 cases of spinal ependymoma and 12 cases of astrocytoma showed that while both *HOXB13* and the other *HOX* family member *HOXA9* were selectively expressed in SP-EPN instead of astrocytoma, only *HOXB13* was expressed exclusively in MPE, whereas *HOXA9* was detected in all subgroups of SP-EPN [12]. This confirms that *HOXB13* can play a role as a potential diagnostic marker, as well as a possible therapeutic target for MPE.

Unlike cranial SEs, how genetic markers influence prognosis in spinal SEs is scarcely studied. For instance, brainstem SEs can have *H3 K27M* mutations without significant effect on prognosis [38], and loss of chromosome 6 and telomerase reverse transcriptase (*TERT*) mutations are frequent events in posterior fossa SE which harbor poorer prognosis [39]. This has yet to be studied in spinal SE. Spinal SE does not appear to be associated with NF2 mutations either.

### 2.2. Ganglioglioma

Gangliogliomas are rare glioneuronal tumors in the spinal cord that resemble their intracranial counterparts but have lower frequency of *BRAF V600E* mutations (two out of nineteen (11%) spinal gangliogliomas were positive for the marker in a case series) [40]. These mutations are less frequent compared to brain tumors but, when present, may offer therapeutic targets [40,41,42]. However, the only confirmed report of a successful treatment of a progressive ganglioglioma with a *BRAF* inhibitor (Vemurafenib) comes from a case report of one patient [42]. Most gangliogliomas are WHO grade I with favorable prognosis following resection. High-grade (anaplastic) variants exhibit increased mitotic activity and cellularity; however, genetic markers specific to spinal cord anaplastic gangliogliomas are not well described in the literature and should be the subject of future research.

### 2.3. Astrocytoma/Glioma

Spinal astrocytomas range from WHO grades II to III (excluding the grade IV glioblastoma) and often harbor heterogeneous and distinct mutations, which does substantially affect the outcome. The histone 3 (*H3*) *K27M* mutations are the most common mutations seen in cerebral astrocytomas, especially diffuse midline gliomas, and affect the histone H3 protein, specifically in the *H3F3A* or *HIST1H3B* genes. They can also occur in spinal astrocytomas and glioblastomas, and data from a study with a sample size of 83 suggest that this mutant could be seen in 40%, 40%, and 20% of grades 2, 3, and 4, respectively [23]. While typically associated with poor prognosis [20,21], recent data suggest grade II/III *H3 K27M*-mutant tumors may have better survival than grade IV astrocytomas, so the histology still has a higher impact on survival than the presence of this mutation per se [22,23].

In contrast to the more common *H3 K27M* mutation, isocitrate dehydrogenase (*IDH*) mutations are relatively rare in spinal astrocytomas and these mutations (*IDH*) are more seen in the cerebral counterparts [23].

Another mutation associated with poor outcome is *TERT* promoter mutations, particularly in *IDH*-wild type (*IDH*-wt) tumors [25,26,27]. However, it is important to note that the prognostic significance of TERT promoter mutations can be modulated by other genetic factors. For instance, patients with these mutations and unmethylated O-6-methylguanine-DNA methyl-140 transferase (*MGMT*) promoters exhibit the worst survival rates, while in *IDH*-mutated gliomas, *TERT* promoter mutations may be associated with a more favorable prognosis, especially in the context of 1p/19q codeletion [24]. Interestingly, *TERT* promoter mutations have a better prognosis in the presence of *K27M* mutations in grades 2 and 3 astrocytomas. Therefore, when considering the prognosis of spine astrocytoma with *TERT* promoter mutations, it is crucial to take into account the combined molecular profile of the tumor rather than solely relying on the presence of *TERT* mutations.

Other mutations with a less clear effect on prognosis include tumor protein 53 (*TP53*) mutations, galanin receptor 1 (*GALR1*) and glutamate receptor 5 (*GRM5*) of neuroactive ligand–receptor interactions signaling pathways, and protein phosphatase 1D (*PPM1D*). While their exact interaction is not well known, they might be associated with a poor outcome and tendency for malignant progression [22,28]. Of note, most these studies have assessed the influence of these mutations in a mixed pool of cranial and spinal astrocytoma and glioblastoma (grades 2–4), with the majority being cranial. Therefore, the generalizability of the conclusions to spine tumors remains questionable, and conclusions should be made only after reassessing the genetic profiling in a pure spinal cord tumor cohort.

Lastly, while effects of *BRAF V600E* mutations on prognosis are still not well known and controversial, their presence may identify candidates for BRAF-targeted therapies (including Vemurafenib, Dabrafenib, and Encorafenib).

### 2.4. Glioblastoma (GBM)

Spinal glioblastomas are exceedingly rare but highly aggressive with dismal progression-free and overall survival rates [43]. Unlike their cerebral counterparts, studies of genetic mutations specific to spinal GBM are scarce, due to rarity of the disease (1–3% of all intramedullary tumors). Moreover, as mentioned previously in Section 2.3, the few studies have a mixed pool of cranial and spinal GBM, making the generalizability even more questionable. Results of the studies have shown that *IDH* (1 and 2) and *ATRX* (Alpha-Thalassemia X-linked Intellectual Disability) mutations and *MGMT* promoter methylation are associated with slightly better prognosis in cranial GBM due to specific treatments for targeting the tumor. In contrast, *TP53* mutations and epithelial growth factor receptor (*EGFR*) amplification are associated with worse outcomes in cranial GBMs. Still, the presence and, as a result, influence of these genetic markers is not well-studied in spinal GBM [44]. Moreover, other studies (again combining cranial and spinal GBMs) have shown that *SPARC* (secreted protein acidic and rich in cysteine) and *VIM* (vimentin) genes are over-expressed and *CACNA1E* (calcium voltage-gated channel subunit alpha1e), *SH3GL2* (SH3 domain-containing *GRB2*-like 2, endophilin A1), and *DDN* (dendrin) genes are under-expressed in glioblastoma. *CACNA1E* and *VIM* exhibit better prognosis. *H3F3A K27M* mutations are common, and co-mutations include *TP53*, *TERT* promoter, and *PIK3CA*. These mutations influence survival and may impact therapeutic choices in GBM in general [45]. To emphasize, these studies often pool cranial and spinal GBM (with cranial ones outnumbering spinal GBM). Therefore, their role in primary spinal GBMs should be selectively studied.

### 2.5. Hemangioblastoma

Spine hemangioblastomas, similar to their cranial counterparts, are strongly linked to mutations in the von Hippel-Lindau (*VHL*) gene, both in inherited (VHL disease) and sporadic cases [46]. While not directly affecting tumor behavior, it may affect outcome due to the higher rate of recurrence (as a result of the syndromic nature of VHL) [32]. While other rare genetic factors may also be involved in cranial hemangioblastoma, particularly in patients with aggressive tumor growth, these are not validated and confirmed in spinal ones.

## 3. Molecular Pathways Altered by the Key Mutations and Their Impact on Spinal Cord Tumors

Understanding how genetic alterations perturb cellular signaling and biology helps bridge genotype to phenotype and clarifies why certain mutations drive aggressive behavior, treatment resistance, or suitability for targeted therapy. These alterations converge on several critical pathways that control proliferation, apoptosis, genomic stability, and epigenetic regulation. Below, the molecular consequences of key mutations/markers—*MYCN* (*N-Myc*), *HOXB13*, *BRAF V600E*, *H3 K27M*, *TERT* promoter, and *TP53*—in the pathogenesis of primary spinal cord tumors (ependymoma, astrocytoma, glioblastoma, and ganglioglioma) are reviewed.

### 3.1. N-Myc/MYCN

*MYCN* is a basic helix–loop–helix leucine zipper transcription factor in the *Myc* family that promotes cell cycle progression, metabolic reprogramming, ribosomal biogenesis, nucleotide synthesis, glycolytic metabolism, and suppression of differentiation programs. *MYCN* amplification leads to massive transcriptional reprogramming through pervasive transcriptional upregulation of proliferative and survival genes and can increase genomic instability. As a result, proliferation is favored, and differentiation is suppressed. *MYCN* overexpression promotes oncogene addiction, meaning the tumor becomes dependent on this single driver for survival. It also induces replicative stress, DNA damage, and genomic instability, accelerating malignant transformation [10,33].

### 3.2. HOXB13

*HOXB13* is a homeobox transcription factor from the *HOX* gene family involved in embryonic patterning and cell identity programs. Aberrant *HOX* gene expression can reflect lineage reprogramming and altered differentiation states. In some tumor types, *HOX* dysregulation is associated with proliferation or changes in developmental transcriptional programs, cell-adhesion, extracellular matrix interactions, and microenvironment interactions. Dysregulated *HOX* gene expression can sustain a stem-like, undifferentiated state and enhance invasive growth. *HOXB13*, in particular, modulates the androgen receptor and *Wnt* signaling [13].

### 3.3. BRAF V600E

*BRAF V600E* is a serine–threonine activating kinase mutation in the *RAS*–*RAF*–*MEK*–*ERK MAPK* pathway. The pathway is essential for inter- and intracellular communication and regulates fundamental cell functions including growth, survival, and differentiation. The pathway also integrates signals from complex intracellular networks in performing cellular functions. The *BRAF V600E* mutation drives *MAPK* pathway hyperactivity by mimicking phosphorylation, which locks BRAF in a constitutively active state and produces a continuous downstream activation of ERK signaling. The result is uncontrolled proliferation, enhanced survival, angiogenesis, and resistance to apoptosis. It also reprograms the tumor microenvironment by upregulating cytokines and growth factors [47].

### 3.4. H3 K27M (H3F3A/HIST1H3B)

Lysine-to-methionine substitutions at *H3 K27* (*H3 K27M*) dominantly inhibit the polycomb repressive complex 2 (*PRC2*) methyltransferase activity, causing global reduction in H3K27 trimethylation (a mark normally associated with transcriptional repression), derepression (i.e., reversing transcriptional repression, resulting in activation of the gene) of developmental programs, and epigenetic reprogramming toward a stem-like, therapy-resistant phenotype. This epigenetic derangement promotes aggressive clinical behavior, therapy resistance, and poor differentiation [48,49].

### 3.5. TERT Promoter Mutations

Activating *TERT* promoter mutations (commonly at C228T or C250T locations) creates new binding motifs for *ETS* (E26 transformation-specific or erythroblast transformation-specific) family transcription factors and up-regulates telomerase reverse transcriptase expression, stabilizing telomeres, and enabling replicative immortality. Elevated telomerase activity prevents telomere shortening, allowing cells to bypass senescence and apoptosis. It also enhances tolerance to oncogenic stress. *TERTp* mutations interact with other driver events to modulate tumor aggressiveness [50].

### 3.6. TP53

*TP53* encodes the tumor suppressor *p53*, the “guardian of the genome”, which is a master regulator of DNA damage responses, senescence, apoptosis, and genomic integrity. Normally, *p53* halts the cell cycle in response to DNA damage, activates apoptosis, and coordinates repair. *TP53* mutation (often missense) or inactivation impairs its DNA-binding capacity, releases constraints on proliferation, increases tolerance to genomic instability, and often cooperates with other oncogenic events to drive aggressive phenotypes. Loss of *p53* function abolishes cell cycle checkpoints, allowing cells with genomic instability to proliferate. It also prevents apoptosis, enabling survival of genetically unstable clones. *TP53* mutations often cooperate with other drivers (*TERT*, *ATRX*, *MYCN*) to accelerate malignant transformation.

In summary, these mutations reshape spinal cord tumor biology by disrupting the hallmarks of cancer: (1) proliferation and cell cycle deregulation (*MYCN*, *TP53*), (2) epigenetic reprogramming and stemness (*H3 K27M*, *HOXB13*), (3) constitutive growth signaling (*BRAF V600E*), (4) replicative immortality (*TERT* promoter). They collectively explain why some spinal tumors demonstrate aggressive, treatment-resistant behavior despite similar histopathological appearances. Although many mechanistic insights are extrapolated from intracranial disease or even other body organ tumors, accumulating spine-specific series increasingly validate the clinical relevance of these pathways in spinal cord tumors.

## 4. Integrating Genetic Data into Clinical Practice

The incorporation of genetic markers into the management of spinal cord tumors marks a significant shift from purely histopathological classification toward a biologically informed approach. Several key domains are impacted: (1) diagnosis, (2) risk stratification, (3) therapeutic decision-making, (4) surveillance, (5) patient and family counseling, (6) research and clinical trials. It should be emphasized that this whole section is based on (author’s) recommendation and currently there is not enough data to support a change in practice. Paradigm shifts in the management of spinal cord tumors has to be implemented only after extensive large multi-center clinical trials.

**Figure 1:**
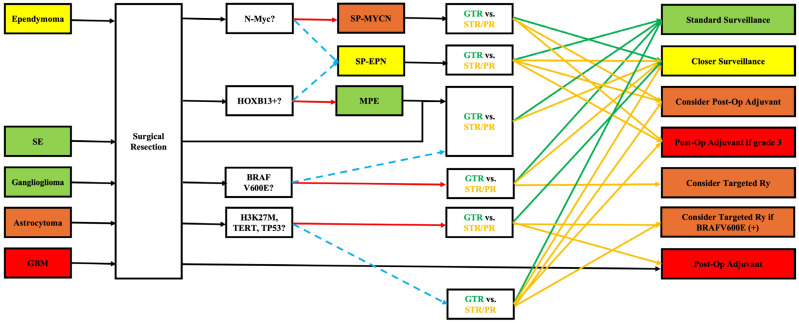
Implementing knowledge from genetic markers into clinical practice. GBM: glioblastoma, GTR: gross total resection, MPE: myxopapillary ependymoma, PR: partial resection, SE: subependymoma, SP-EPN: spinal ependymoma, SP-MYCN: spinal ependymoma with *N-Myc* amplification, STR: subtotal resection. Red solid and blue dashed arrows signify positive and negative answers, respectively. Green and yellow solid arrows signify next steps for GTR and STR/PR, respectively.

DNA methylation profiling and gene fusion detection have proven more reliable than histological grade alone in predicting prognosis. For example, the identification of *MYCN* amplification in spinal ependymoma or *H3 K27M* mutations in astrocytoma can guide clinicians toward more aggressive surveillance and treatment plans, even when traditional features suggest a low-grade lesion. Moreover, while surgery remains the cornerstone for most primary intramedullary spinal cord tumors, molecular data inform decisions about adjuvant therapy. For instance, while prognosis in ependymoma is generally favorable and achieving gross-total resection (GTR) is technically considered a “cure” without further treatment, identifying *N-Myc* amplification can change the standard classification to SP-MYCN bearing a worse prognosis. In these scenarios, the multi-disciplinary treatment team (including the surgeon, radiation oncologist, and medical oncologist) can decide on more frequent imaging surveillance if GTR is achieved; if sub-total resection (STR) or partial resection (PR) is achieved, adjuvant treatments (post-operative radiation +/− chemotherapy) may be considered as an earlier option (instead of waiting until recurrence happens). Likewise, identification of *HOXB13* may not alter the treatment strategy (with GTR still being the goal) but confers better prognosis as it confirms diagnosis of MPE (vs. SP-EPN). For astrocytoma and GBM, the genetic mutation profile (mostly comprising *H3 K27M*, *TERT*, and *TP53*) can significantly alter (worsen) the prognosis. Again, the treatment plan has to be modified to implement more frequent clinical and imaging surveillance and more aggressive treatment modalities (surgical resection, radiation, chemo, etc.). In some instances, specific markers do not alter the prognosis, but they can guide toward additional beneficial treatments. For instance, the presence of *BRAF V600E* mutations, particularly in gangliogliomas or astrocytomas, offers potential access to targeted inhibitors (e.g., vemurafenib, dabrafenib, and encorafenib), shifting paradigms for recurrent or unresectable disease. Genetic testing also provides value in familial syndromes and counseling. Detection of NF2 mutations in ependymomas mandates genetic counseling and long-term follow-up for other manifestations of neurofibromatosis type 2. Lastly, as the information is relatively new and its impact on clinical practice still not fully established, patients with defined alterations may be eligible for molecularly stratified clinical trials, enabling more tailored medicine approaches in neuro-oncology for spinal tumors. A schematic workflow is proposed and depicted in Figure 1. Of note, as previously mentioned, the field is still fresh, and most data originates from case series. Therefore, the author needs to emphasize that the whole review and suggested workflow is purposed to increase awareness about the topic and open door for future research. Practical changes and guidelines can only be concluded based on extensive multi-center large clinical trials.

## 5. Limitations and Future Directions

Despite its promise, the integration of genetic diagnostics into routine clinical workflow for spinal cord tumors faces several challenges.

First, most genetic tests are available only in certain labs and settings and their availability is relatively limited in most practices, including community settings. Other access barriers include the high cost of most genetic testing (including NGS and methylation profiling), limited insurance coverage, and the need for specialized infrastructure, which collectively restrict widespread adoption outside of tertiary centers. Furthermore, quality assurance remains a concern: variability in assay platforms, sequencing depth, and interpretation algorithms can lead to inter-laboratory discrepancies in reported results. Even when the same test is performed, subtle differences in analytic pipelines or reference databases may alter the classification of a tumor or the detection of a pathogenic variant. Altogether, in CNS-wide benchmarking, inter-laboratory concordance is typically only ~85–90% for methylation profiling and NGS panels [51,52,53]. Standardization of protocols, proficiency testing, and centralized registries are therefore essential to ensure reproducibility and reliability of molecular diagnostics across institutions. To overcome these barriers, emerging technologies such as digital pathology and artificial intelligence (AI) are promising features to reshape the landscape of molecular diagnostics. AI applied to whole-slide images can detect subtle morphologic correlates of key mutations (e.g., *H3 K27M*, *BRAF V600E*) and may triage cases for molecular testing, although reliability for rarer spinal tumors is limited by small datasets and inter-laboratory variability. These tools hold promise as adjuncts to streamline workflows but remain as a form of decision-support rather than standalone diagnostic modalities. Furthermore, liquid biopsy, particularly analysis of cerebrospinal fluid (CSF), offers a minimally invasive route to detect tumor-derived DNA. Hotspot mutations such as *H3 K27M*, *BRAF V600E*, *TERT* promoter, and sometimes *TP53* can be assayed from CSF using droplet digital polymerase chain reaction (ddPCR) or ultra-deep NGS with sensitivities reported in the 70–90% range in CNS cohorts. In contrast, *MYCN* amplification and *HOXB13* expression are not yet reliably detected from CSF [54]. Current utility lies in patients where biopsy is unsafe or for monitoring recurrence, while broader applications such as methylation profiling or comprehensive copy-number analysis from CSF remain experimental. Near-term clinical adoption is therefore the most realistic for hotspot mutation detection, whereas AI-based digital pathology and advanced liquid biopsy applications require further validation before routine use in spinal cord tumors.

Secondly, given the rarity of intramedullary spinal cord tumors, studies are faced with rather small sample sizes which restrict robust validation of markers. Adding to the limitation and as noticeable in this review, most data stems from case series, rather than large clinical trials. Although data is more readily available about the cranial counterparts, the rarity of primary intramedullary spinal tumors have refined the generalizability of the data. This issue also imposes challenges on prospective clinical trials which are trying to define the impact of genetic subtypes on treatment strategies and outcomes.

Lastly, overlapping genetic profiles in some tumors complicate classification. While the effects of one single mutation on outcome can be better evaluated, presence of multiple genes and genetic mutation can alter the interaction due to polygenic inheritance, epistasis, genetic linkage, etc., complicating the clinical outcome (for instance, as observed in the case of *TERT* promoter mutation and *H3 K27M* in astrocytomas). To overcome these limitations, collaborative registries and standardized panels for genetic testing are needed.

Despite all the restrictions and limitations, the main goal of the current review is to bring awareness about the importance of genetic marker testing in primary spinal cord tumors and encourage practitioners and researchers to consider and incorporate genetic testing in this disease population. Accordingly, at the author’s institution, efforts are made to implement routine genetic testing for all primary spinal cord tumors. Only increasing the case volumes and larger clinical trials with adequate follow-up can prove (or disapprove) the importance of the topic and make necessary paradigm shifts in intramedullary tumor care. The controversial questions that need to be answered in tumors with unfavorable genetic profiling are whether or not: (1) radiotherapy is to be added to surgical resection after a GTR; (2) radiotherapy details are be changed (i.e., starting time, number of sessions, dose, etc.), especially in cases of STR or PR (3) patient surveillance schedule is to be altered because the risk of recurrence is expected to be higher. The main purpose of the current review is to open discussion and pave the way for more detailed clinical trials and in-depth research efforts. The final goal is to demonstrate how new information on genetic profiling can change our current practice and blend into the data regarding tumor pathology, tumor grade (especially in astrocytoma), and extent of resection.

## 6. Conclusions

Advances in genetic profiling have reshaped our understanding of primary spinal cord tumors. Rather than relying solely on histopathology, clinicians can now stratify patients by genetic alterations, improving prognostication and treatment selection. For instance, *N-Myc* amplification changes the classification and worsens the prognosis of spinal cord ependymoma, as do *H3 K27M, TP53*, and *TERT* promoter mutations, among many, worsen the prognosis of astrocytomas. Moreover, *BRAF V600E* can potentially serve as a therapeutic target in ganglioglioma and astrocytoma. As such, molecular markers are not merely academic observations; they are actionable elements that can and should inform patient care. As the field evolves, it is imperative that neuro-oncology practice transitions from morphology to genetic and molecular precision, with the ultimate goal of enhancing survival and quality of life for patients with these rare but impactful tumors.

## Figures and Tables

**Table 1:** Genetic markers in different intramedullary spinal cord tumors and their association with prognosis. NR: Not reported (when median survival in months was not acknowledged through the literature search). WT: Wild-type.

*Tumor Pathology*	*Tumor Subtype*	*Grade*	*Marker*	*Prognosis (Compared to WT)*	*Median Progression-Free Survival (Months)*	*Median Overall Survival (Months)*	*References*
Ependymoma	Spinal	2–3	WT	Good	82	180	[7,8,9]
	Spinal with N-Myc		*N-Myc*	Worse	17	87	[10,11]
	Myxopapillary	2	*HOXB13*	Good—has diagnostic value	82	180	[12,13]
	Subependymoma	1		Good	NR	NR	[14]
Ganglioglioma		1	*BRAF V600E* (rare)	Good, mutation may have therapeutic value	67	NR	[15,16]
Astrocytoma		2–3	WT	Fair		96–48	[17,18,19]
			*H3 K27M*	Worse	3–14	15 (5–21)	[20,21,22,23]
			*TERT*	Worse	7–9	14.6–22	[23,24,25,26,27]
			*TP53*	Worse	NR	11.5–30	[22,28]
			*GALR1*	Unclear, most likely worse	NR	NR	[22,28]
			*GRM5*	Unclear, most likely worse	NR	NR	[22,28]
			*BRAF V600E*	Unknown, may have therapeutic value	NR	NR	[22,28]
			*PPM1D*	Unclear, most likely worse	NR	NR	[22,28]
Glioblastoma		4	WT	Poor	6–11	13 (10–21)	[29,30,31]
			Unknown		NR	NR	
Hemangioblastoma		1	*VHL*	Slightly worse due to higher recurrence	NR	NR	[32]

## Data Availability

All data discussed are derived from published studies cited in the manuscript.
